# Supplementary material for: The coevolution of contagion and behavior with increasing and decreasing awareness
Source: PLoS One. 2019 Dec 3;14(12):e0225447. doi: 10.1371/journal.pone.0225447 (PMC6890210; doi:10.1371/journal.pone.0225447)
Supplement: Attachment — Submitted filename: Response_Reviewers_PONE-D-19-20285.pdf [file pone.0225447.s001.pdf]

Comments from the Academic Editor

Comment 0.1

Please ensure that your manuscript meets PLOS ONE's style requirements, including those for file naming. The PLOS ONE style templates can be found at `http://www.journals.plos.org/plosone/s/file?id=wjVg/PLOSOne_formatting_sample_main_body.pdf` and `http://www.journals.plos.org/plosone/s/file?id=ba62/PLOSOne_formatting_sample_title_authors_affiliations.pdf`

Reply

We have checked the PLOS ONE style as required by the Academic Editor. The LaTeX style was the prescribed one (plos2015.bst) and the paper was written by adapting the PLOS ONE LaTeX template. We did not change any preset configuration, only added some packages. We have also checked the paper for compliance with the guidelines pointed out by the AE. If any requirement still results unmet, it escaped our double check and we apologize for that.

Comment 0.2

Our internal editors have looked over your manuscript and determined that it is within the scope of our Science of Stories Call for Papers. This collection of papers is headed by a team of Guest Editors for PLOS ONE: Peter Sheridan Dodds, Mirta Galesic, Mohit Iyyer, and Matthew Jockers. Additional information on this Call for Papers can be found on our website: https://collections.plos.org/s/science-of-stories. Please note: Sharing the data underlying the findings will be a requirement of publication, per the PLOS data policy. As applicable, under this Call for Papers, authors are expected to provide, upon submission, the source code needed to replicate their findings, ideally in a repository (such as Zenodo, which can also import from GitHub) or a suitable cloud computing service (such as Code Ocean). Please specify whether or not you would like your manuscript to be considered for the collection in your cover letter.

Reply

Thank you. We were extremely glad to know that our paper could be considered for the Science of Stories Call for Papers.

With regard to the sharing of data and code, we have done what required. The details are in the answer to the next comment, which is specific of this aspect.

Comment 0.3

3. In order to meet the requirements for the Science of Stories collection, the Guest Editors ask that you please make the code to reproduce your analysis available in a stable, public repository (for example, Zenodo, or GitHub) or a suitable cloud computing service (such as Code Ocean) when submitting your revised manuscript. The code should include a license file and detailed readme so that someone with access to the dataset is able to reproduce your analysis using the code. We ask that you include the DOI for the repository holding your code in an updated Data Availability statement with your revised manuscript.

Reply

We have set up a GitHub repository (`https://github.com/mc-unimi/epidemics-awareness-paper`) that contains both the code of the multiagent epidemic simulator (folder `code`) and the data as CSV files (folder `data`). A v1.0 release has been created and synchronized with Zenodo. The DOI is **10.5281/zenodo.3462521** (`http://doi.org/10.5281/zenodo.3462521`) and we included it in the Data Availability statement.

The data is of two types:

- CSV files used to produce Figure 3, 4, 7, and 8 based on average results of the simulation trials;
- CSV files containing all valid trials (subfolder `trials`) from which the averages have been computed.

With this resubmission, each data point of Figures 3, 4, 7, and 8 is averaged over, at least, 200 valid trials, to comply with Reviewer 2's Comment 2.3.

For the code we included the GPLv3 license; data is licensed under the Creative Commons Attribution 3.0 Unported license. In all folders a README file explains the content. The `code`'s README contains a brief guideline to run the simulator. Other comments have been included into the source code. The README in the `trials` subfolder has a detailed explanation of the naming convention we used for the trials, which should help identifying to which figure and configuration the results refer to. In addition, we uploaded a table summarizing all parameters of the simulator and the settings used for the trials (`Parameters_Configuration_Chart.pdf`).

Comments from Reviewer 1

Comment 1.1

The coevolution spreading of epidemic and awareness on networks is a hot topic in recent years. In this paper, the authors studied the epidemic spreading with awareness, and found some interesting phenomena. I want to recommend its publication in PLoS ONE. I have a few minor comments.

Reply

Thank you. Your comment was extremely rewarding for us.

Comment 1.2

The coevolution of epidemic and awareness is a hot topic and closely related to this work. The author should discuss more, and some related papers are https://doi.org/10.1016/j.physrep.2019.07.001; Scientific Reports 6 (2016) 29259; Scientific Reports 4 (2014) 5097.

Reply

Reviewer 1's suggestion was precious, in particular the first paper "Coevolution spreading in complex networks", a large survey appeared right before our submission, is a trove of relevant work and has been extremely useful for our revision. We expanded our discussion of the coevolution of epidemic and awareness citing several new papers and more clearly presenting our research goals. How we aimed at contributing to the

research on epidemic and awareness has also been clarified, pointing out the different approach we have adopted with respect to previous works. In particular, we have stressed out the case of coexisting positive and negative social reinforcements that may play an important role in disease-behavior models, especially when the epidemic vector is not a disease. To the best of our knowledge, ours is one of the few works explicitly tackling with this scenario. This has suggested us to reconsider the expression "disease-behavior" and propose "contagion-behavior" as a more general classification and possibly better suited for systems and networks studies. The new references have also helped in improving the model description and discussing its limitations. More details about the model description are discussed in the reply to Reviewer 1's Comment 1.4.

To this end, we have rewritten a large part of the Introduction by using, as the main references, the papers mentioned in Reviewer 1's comment and others relevant we have found following their citations. Now the work is presented as belonging to the general class of disease/behavior models (with the addition of the proposed new definition) and introduced explaining the coevolving dynamics between contagion and agents' behavior. We have pointed out one of the novelty we believe our model is presenting, namely the positive and negative variation of awareness, without assuming memory loss long-term effects. The new text is in the Introduction, annotated as R1.2.

Comment 1.3

I think the evolution mechanisms of the three different dynamics disease, addiction, and rumors are similar, that is they are simple contagions. Once the contagion with social reinforcement, I think the situation is different. Therefore, the authors should be specific that their research object is simple contagions and discussed what will happen for complex contagions in the discussion section.

Reply

With respect to the distinction between simple and complex contagion, Reviewer 1 is right commenting that the underlying dynamics is the same for the three categories we have considered and it is a traditional simple contagion: every contact between a Susceptible and an Infected is independent and has equal probability to induce a change of state. However, the situation is different when we consider the awareness dynamics we have modeled and then how state transition probabilities are modified. For awareness, thresholds are always in place in order to trigger the imitation behavior (positive for the disease class, positive and negative for the addiction category, positive, negative, and through direct messages for the rumor class). Another non linear effect is given by the logistic function correlating awareness changes with changes of infection and recovery probabilities. This represents, we guess, a form of *indirect* complex contagion dynamics, because multiple exposures to different neighbors are required to reach the imitation/message thresholds that trigger a change in awareness, which then induces a (non-linear) change in state transition probabilities. Therefore, to us our model looks like a mixed (or hybrid) model with both simple and complex contagion effects: *i)* simple contagion as the simplified assumption for the base dynamics; and *ii)* a complex contagion effect for dynamic changes of base rates (infection and recovery probabilities) driven by the corresponding adaptation, positive or negative, of awareness triggered by social reinforcement.

A more realistic model would have included a complex contagion dynamics for state transition, too, this way combining two layers of social reinforcement: one for awareness dynamic adaptation and another for infection and recovery, possibly with different

regimes (under some assuptions awareness dynamics could be regressive with respect to epidemic effects, in other cases it could be the opposite, with strong hype concerning the effects or extent of an epidemic). We guess that a study about the coevolution of these two dynamics, represented as both based on social reinforcement and, for the awareness, including both positive and negative effects could be a difficult but challenging research goal for future works.

In the paper, we introduced a discussion similar to what written here in the subsection "" of the new section "Awareness, imitation, and contagion dynamics" (see the reply to the next comment).

The subsection "Awareness, imitation, and contagion dynamics" is annotated as R1.3 For better readibility, we did not marked in red the text of the whole subsection, but only the title.

Comment 1.4

The model description is not very clear, and please improve it.

Reply

Thanks also to the new references, we have improved the model description by discussing: *i)* the hybrid contagion characteristics (simple for the base dynamics; complex for the dynamic changes to base rates); *ii)* the awareness dynamics in an heterogeneous population; and *iii)* how we adapted a threshold model to positive and negative social reinforcements. We have also improved the discussion about the limits of our model that, if on the one side aims at introducing a novelty in disease-behavior systems studies, on the other it comes with clear limitations.

Part of the improvement was done by removing from the model section all parts not specifically related to its description. We have added a new section after the Introduction titled "Awareness, imitation, and contagion dynamics". This new section now should contain all parts, previously scattered in different sections of the paper, related to concepts and proposed solutions about awareness and its factors, the epidemic categories and positive-negative imitations mechanisms.

From the model section, we moved most of the previous "Multicomponent awareness" subsection, which presented definitions of awareness contributing factors (including Table 1), the discussion regarding Imitation and messages, and the definition of the reference epidemic categories. This way, the model section should be more specific.

Section "The model" has been largely reorganized and rewritten to respond to Reviewer 2. We forward Reviewer 1 to our reply to Comment 2.4 for more details.

The new section "Awareness, imitation, and contagion dynamics" is annotated as R1.4 and R2.4 to refer to Reviewer 2's comment, which was dissatisfied with the section "Multicomponent awareness", which contained part of the content moved here. For better readibility, we did not marked in red the text of the whole section, but only the title and the paragraphs completely rewritten.

Comment 1.5

The resolution of the figures is meager, and please improve them.

Reply

We apologize for this carelessness, also Reviewer 2 expressed her/his comprehensible disappointment for the problem with figures' quality. Original image files were clear and

of good resolution, but during the submission the pdf conversion blurred them. The Corresponding Author knew that it may happen and the submission should be double-checked, he takes the full responsibility for this inconvenience. With this resubmission, the problem should be solved, in addition most figures (i.e., Fig. 3,4,5,8,9) have been plotted again based on the enlarged set of trials, as for Reviewer 2's Comment 2.3.

Comment 1.6

Add some applications about the results.

Reply

In the previous submission only a short paragraph in the Introduction and another short paragraph in the Conclusions mentioned possible applications. Now we have moved the whole discussion to the Conclusions section and extended it. We have presented the case of *awareness campaign planners* (e.g., public policy makers, educators) and some of the common problems they face to. Two, in particular, are the applications we envisioned. One is about the outcome of a real awareness campaign, which is most often a combination of an immediate benefit (individuals increase their awareness), a possible network benefit (more aware individuals may act as social reinforcement for the peers), but also of individuals that quickly lose their awareness due to adverse social reinforcement in their social context. Our model is a first attempt to account for all these combined effects. The second application is for supporting decision makers in the selection of the target of an awareness campaign. A difficult choice is between a non negligible improvement in awareness distributed over a large group of unaware individuals or a focused intervention over a smaller group of individuals aimed at making them well aware. Again, our model is an attempt at supporting such a decision. We have also added, in the Conclusions too, a final paragraph about the limitations of our model, which aims at proposing some original solutions, but it is limited in several ways. You will find the new text in the Conclusions annotated as R1.6.

Comments from Reviewer 2

Comment 2.1

The figures are too messy and too blurred.

Reply

We completely understand the disappointment for the problem with figures' quality, also Reviewer 1 expressed it. This was due to carelessness for which the Corresponding Author takes the full blame. Original image files were clear and of good resolution, but during the submission the pdf conversion blurred them. He knew that it may happen, he had to check. With this resubmission, the problem should be solved, in addition most figures (i.e., Fig. 2,3,4,7,8) have been plotted again based on the enlarged set of trials, as for Comment 2.3.

Comment 2.2

Too many experimental parameters are not given.

Reply

We have largely reviewed the initial part of the Result section, also in response to the next Comment 2.3, and included a part presenting more explicitly and clearly the parameters used in simulations. We also added a new table (Table 2, ”Configuration values for different groups of simulations”) with the short description and value of each parameter for the three groups of experiments. The annotation about modifications referred to this comment is R2.2.

Comment 2.3

The authors’ workload is insufficient, for example, too few experiments were repeated which bring about the messy lines in the figures.

Reply

The reviewer was absolutely right, the number of trials for calculating average data points had to be considerably increased; by doing again all experiments the results appear with more clarity. All results showed in the plots improved, but Fig.5 did it in a particularly way, we believe, both in clarity and in quality of the result, being a spurious result that appeared in the previous version, due to the insufficient number of experiments, now absent. With this resubmission, at least 200 valid trials have been produced for each average data point plotted in Fig.4/5/8/9. Valid trials have been considered those that propagated the contagion at least for 3 time steps and at least infecting 50 nodes (5% of the total population size, N=1000). These two conditions have been set empirically given the fact that: *i)* with 3 time steps many simulations comes close to the peak of infected nodes; *ii)* the total of 50 infected nodes is sufficient to discard the (rare) cases where the contagion seemed to jump back and forth for several time steps among few nodes. Given the reference configuration that we used throughout our simulations, with an infection probability $P_{SI} = 0.2$, a recovery probability $P_{IR} = 0.5$, and the presence of only a single seed node, the large majority of non-valid trials corresponded to simulations that terminated within the first two time steps and with a number of infected nodes much smaller than 50. For each unique configuration, the total number of simulations we run was between 800 and 1000, including valid and non-valid ones. For example, Fig.4, which compares 8 configurations, is now the result of more than 6000 simulations.

We found a related work to confront our validity criteria. In [1], ”an outbreak is defined as a minimum final epidemic size of 25 (i.e. 0.5% of the total population size N=5000)” (page 3, caption of Fig. 2). Infection and recovery probabilities are both equal to 0.1 in their stochastic simulations, and in total they run 10000 simulation for unique network. Given the different network sizes and probabilities, it seems to us that our criteria to consider a trial as valid is comparable to the one assumed in [1].

We have included this explanation in Section ”Results”, annotated as R2.3.

Comment 2.4

In the section multicomponent awareness, the authors described a lot of useless content.

Reply

We have removed from subsection ”Multicomponent awareness” the parts discussing components not implemented in the simulations and then not relevant for the results of this paper. The former subsection ”Multicomponent awareness” has been rewritten like

several other parts of the section ”The model”. The whole section ”The model” has been reorganized to improve clarity and completeness. A new subsection titled ”Schema of the coevolution dynamics” has been added to describe in a schematic way the coevolution between contagion and awareness dynamics. A new figure (now Fig. 2) was also added to ease the model comprehension. For readibility, we have marked in red only the titles referring to sections with copious edits and the paragraphs completely new. The annotation about modifications referred to this comment is R2.4.

Comment 2.5

The logic between experiment results and the conclusions is not strong.

Reply

The presentation of experiment results has been reorganized in many parts, several paragraphs rewritten. This, together with the general revision of the whole paper, has informed a complete rewriting of the Conclusion section. Now it seems to us more pertinent and clear in the logic, with respect to the results, with references to possible applications, and to some of the limitations of the work.

The annotation about modifications referred to this comment is R2.5.
